# Molecular predictors of recurrence in vestibular schwannomas: a transcriptomic and RT-qPCR analysis

**DOI:** 10.1007/s00405-026-10548-7

**Published:** 2026-08-26

**Authors:** Carmen Ruiz-García, Sandra Franco-Caspueñas, Lourdes Rodríguez-de la Rosa, Victor Martinez-Glez, J. Manuel Morales-Puebla, Isabel Varela-Nieto, Luis Lassaletta

**Affiliations:** 1 Department of Otorhinolaryngology, La Paz University Hospital, Paseo de la Castellana 261, 28046 Madrid, Spain; 2 Neurobiology of Hearing and Myelinopathies, Institute for Biomedical Research Sols-Morreale (IIBM), CSIC-UAM, Arturo Duperier 4, 28029 Madrid, Spain; 3 Research in Otoneurosurgery, Hospital La Paz Institute for Health Research - IdiPAZ, Paseo de la Castellana 261, 28046 Madrid, Spain; 4 PhD Program in Medicine and Surgery, Faculty of Medicine, Autonomous University of Madrid, Arzobispo Morcillo 4, 28029 Madrid, Spain; 5 Biomedical Research Networking Centre On Rare Diseases (CIBERER), Institute of Health Carlos III, Monforte de Lemos 3-5, 28029 Madrid, Spain; 6 Department of Biology, Autonomous University of Madrid, Darwin 2, 28049 Madrid, Spain; 7 Institute of Medical and Molecular Genetics, INGEMM-IdiPAZ, La Paz University Hospital, Paseo de la Castellana 261, 28046 Madrid, Spain; 8 Center for Genomic Medicine, Parc Taulí Hospital Universitari, Institut d’Investigació i Innovació Parc Taulí (I3PT-CERCA), Plaça Torre de l’Aigua, 08208 Sabadell, Spain

**Keywords:** Acoustic neuroma, NF2-associated schwannomatosis, Tumor biology, Gene expression profiling, Interleukin-8, Tumor necrosis factor

## Abstract

**Purpose:**

Vestibular schwannomas (VS) are benign tumors with variable clinical behavior. Recurrence after surgery is uncommon yet sometimes aggressive. Reliable molecular predictors of recurrence are lacking, and the correlation between genotype and phenotype is weak. This study aimed to identify molecular markers associated with recurrence in VS and explore their prognostic potential.

**Methods:**

Retrospective case-control study of patients undergoing VS surgery between 2017 and 2024. RNA-Seq was conducted to identify molecular signatures associated with recurrence, and selected genes were validated using RT-qPCR. Recurrence-free and salvage surgery-free survival were analyzed by the extent of resection.

**Results:**

23 samples from 22 patients were analyzed, including 7 recurrent and 16 non-recurrent cases. 81 differentially expressed genes were identified in recurrent tumors, with enrichment of the MAPK (FDR = 7.9 × 10⁻⁴) and PI3K/AKT/mTOR (FDR = 0.038) pathways. Eight candidate genes were selected for validation: *DCN* (FC 4.34); *DUSP5* (FC 4.20); *CXCL8* (FC 3.25); *JUND* (FC 3.27); *MYC* (FC 2.45); *MAFF* (FC 3.79); *SOD3* (FC 2.47) (all upregulated), and *TNF* (FC 0.16) (downregulated). RT-qPCR confirmed that *CXCL8* and *TNF* were significantly elevated in tumors requiring early salvage surgery and in an NF2-associated Schwannomatosis case. No differences in recurrence-free or salvage surgery-free survival were observed between near-total and subtotal resections.

**Conclusion:**

Recurrent VS exhibit a distinct molecular profile, highlighting IL-8 and TNFα as potential biomarkers and therapeutic targets. These findings support the development of a prognostic panel to guide personalized follow-up and treatment, particularly for high-risk patients. Further studies are needed before clinical implementation.

## Introduction

Vestibular schwannomas (VS) are benign and typically slow-growing tumors that originate from the Schwann cells of the vestibulocochlear nerve [1]. While most cases are sporadic, approximately 5% are associated with NF2-related Schwannomatosis (NF2-SWN), a rare systemic disorder [1, 2].

Treatment is highly individualized, depending on tumor characteristics and patient factors. For growing or symptomatic tumors, treatment decisions depend on tumor size, patient age and comorbidities, degree of hearing loss, and other symptoms such as facial nerve dysfunction [3].

Surgical strategy depends on tumor size and preoperative hearing status. In the past, surgeons aimed for complete tumor resection to prevent recurrence and avoid reoperation. However, current surgical philosophy prioritizes patient quality of life over radical resection, aiming to minimize morbidity [4–6].

The extent of resection (EOR) is typically classified as gross total resection (GTR), near-total resection (NTR), or subtotal resection (STR). The distinction between these categories remains inconsistently defined in the literature [7, 8]. However, NTR generally refers to leaving a small tumor remnant adherent to a central neurovascular structure, which is not detectable on postoperative imaging [9], or to resection of > = 95% of the tumor volume [5, 10]. In contrast, STR is defined as leaving a visible residual tumor on imaging or removing < 95% of the tumor volume.

GTR typically results in no detectable residual tumor. In most cases of NTR or STR, residual tumor remains stable [10, 11]. Some patients, however, experience recurrence, defined either as evidence of a new tumor following GTR or progression after NTR or STR [8].

Reported recurrence rates are likely unreliable and underestimated due to inconsistent follow-up duration and heterogeneous reporting of EOR [10, 12]. Long-term studies indicate 10-year recurrence-free survival ranging from 87% for GTR to 73% for STR. However, there is no significant difference between NTR and STR when analyzing the need for salvage therapy [10].

The molecular mechanisms underlying recurrence remain poorly understood. Emerging evidence suggests an association between increased immune reactivity and recurrence [13], including increased macrophage and CD4+/CD8 + T cell densities and a higher molecular immunology borstel index [12]. Additionally, it has been hypothesized that alterations in the tumor microenvironment [14, 15] may also influence the biological behavior of the residual tumor [9]. Transcriptomic analysis has demonstrated that rapidly progressing tumor remnants exhibit differential expression of immune-related genes, implicating T-cell immunosenescence in tumor progression [13].

Building on prior evidence, we hypothesize that specific molecular alterations distinguish recurrent from non-recurrent VS. Since reliable molecular predictors are lacking, our objective is to identify distinct markers associated with recurrence and the need of salvage surgery. We ultimately aim to develop a prognostic panel to help identify high-risk cases, support personalized follow-up, and enable earlier interventions.

## Methods

### Clinical data analysis

Included patients underwent lateral skull base surgery between 2017 and 2024, with a confirmed diagnosis of VS by a senior pathologist. Demographics and clinical variables included age at diagnosis, sex, radiologic features (homogeneous, heterogeneous, and cystic) [16], previous treatments (radiotherapy or surgery, including EOR), and diagnosis of NF2-SWN. Hearing was classified into four grades (A-D) according to the American Academy of Otolaryngology-Head and Neck Surgery [17, 18]. The senior surgeon assessed EOR at the end of the surgery and classified it as GTR, NTR, or STR, as previously defined.

Every case of recurrence or tumor growth prompting salvage surgery during the study period was included as a recurrence, totaling 7 cases. Non-recurrent patients were considered controls. Recurrence was further classified into aggressive and indolent. The “aggressive” (AR) subgroup included patients with early recurrence (occurring within 2 years of follow-up) who required salvage surgery, and NF2-SWN recurrence patients. In contrast, the “indolent” recurrence (IR) subgroup comprised patients with late recurrence (defined as after 2 years of follow-up) requiring surgery. 

### Survival analysis

Survival analysis for recurrent tumors utilized two temporal metrics. The recurrence-free interval was defined as the number of months between the primary surgery and either significant growth documented on MRI or the last MRI, if no radiological progression was observed. The salvage surgery-free interval was defined as the number of months from the initial surgery to the first salvage surgery, or until the last control in patients who did not require further surgery. The primary outcomes were recurrence-free survival (RFS) and salvage surgery-free survival (SSFS). 

### Surgical procedure and tissue sample handling

All patients underwent a translabyrinthine approach except for one case (VS52), in which a transcochlear approach was utilized. During surgery, tissue samples were collected from non-coagulated areas, preserved in RNA*later* (Thermo Fisher Scientific) stabilization solution, and immediately transported on dry ice to be stored at -80 °C until further processing. 

### Genetic analysis

Following surgery, 18 patients underwent evaluation to detect *NF2* gene alterations. Genetic analysis was performed on blood and/or tissue samples, as available. Next-generation sequencing was performed in every case. Peripheral blood DNA was extracted using a magnetic-separation method with the Chemagic 360 or Chemagen Magtration System 8Lx (PerkinElmer). DNA from paraffin-embedded tissue was extracted using the TruXtrac FFPE kit (Covaris). DNA from fresh tissue was extracted using the High Pure PCR Template Kit (Roche). Until 2022, DNA isolation was followed by targeted gene capture using the OftSd-v1-1 custom panel. From 2022 onward, Whole Exome Sequencing was performed using the Illumina HiSeq4000 or NovaSeq6000 platform. Variant classification was conducted according to the American College of Medical Genetics and Genomics guidelines [19]. 

### Transcriptomic and bioinformatic analysis

Total RNA was extracted from 10 VS tumor samples. Samples were processed using QIAzol Lysis Reagent (Qiagen) on the QIAcube automatic station according to the manufacturer’s protocol.

RNA integrity was assessed using the Agilent 2100 Bioanalyzer (Agilent Technologies), and concentration was measured using the Qubit RNA Broad Range Assay Kit (Thermo Fisher Scientific). Samples with RNA Integrity Number > 7 and concentration > 20 ng/µL were selected.

RNA sequencing libraries were prepared using the NEBNext Ultra II Directional RNA Library Prep Kit for Illumina (New England Biolabs), following the manufacturer’s protocol. Sequencing was performed using the Illumina NextSeq 500 platform (Illumina, Inc.), and raw data were generated in FASTQ format, suitable for downstream bioinformatic analysis. Reads were aligned to the human reference genome using Spliced Transcripts Alignment to a Reference. Variant annotation and population frequency analysis were conducted using the Genome Aggregation Database. All sequencing was carried out at the Genomics Unit, Madrid Science Park Foundation.

Differentially expressed genes (DEGs) were identified by comparing recurrence cases (*n* = 4) to the controls (*n* = 6). Genes with a fold change (FC) > 2 or < 0.5 were selected for further analysis. Protein-protein interaction networks between DEGs were explored, along with pathway enrichment analysis, to identify clinically relevant pathways involved [20]. The STRING tool and EnrichmentMap application [21] (Cytoscape 3.10.3) were used for network construction and analysis. 

### RT-qPCR validation

Total RNA extraction and quantification of additional 11 samples were performed using the RNEasy Mini Kit (Qiagen) and Qubit RNA Broad Range Kit (Thermo Fisher Scientific), respectively. 500ng of RNA from each reaction was used for cDNA synthesis, using iScript cDNA Synthesis Kit (Bio-Rad). Quantitative PCR was conducted using FS SYBR Green Master Mix (Applied Biosystems) or Green Master Mix (Nzytech) with specific primers in a CFX96 Touch Real-Time PCR System (Bio-Rad). Gene expression levels were normalized to *18 S*,* TBP* and *HPRT*. Relative expression of genes of interest was calculated using the 2^(−ΔΔCT)^ method. The list of analyzed genes is provided in Table 1. Primer sequences are available upon request.

**Table 1:** Selected genes for RT-qPCR validation. Summary of eight differentially expressed genes selected for RT-qPCR validation after RNA Sequencing analysis, including their fold-change (FC) values, statistical significance (q-value), key biological functions, and any prior associations with Vestibular Schwannoma (VS). All genes were significantly dysregulated (FC > 2/<0.5; q < 0.05)

Gene name	FC	q-value	Protein function	Previously studied in VS
*DCN* (Decorin)	4.34	0.03	Facilitates collagen fibril assembly. Complex role in tumorigenesis, inhibiting angiogenesis and proliferation and promoting autophagy and inflammation [22].	No
*DUSP5* (Dual specificity phosphatase 5)	4.20	0.02	Negatively regulates ERK signaling within the MAPK (mitogen-activated protein kinase) family, which is associated with cell proliferation and differentiation [23].	No
*CXCL8* (C-X-C motif chemokine ligand 8)	3.25	0.05	Interleukin-8 (IL-8). Secreted by several inflammatory and tumor cells. Plays a major role in the inflammatory response. It also promotes angiogenesis, invasion, and metastasis [24].	No
*JUND* (Jun D proto-oncogene, AP-1 transcription factor subunit)	3.27	0.02	Protects cells from apoptotic response to stress [25]. Binds enhancers of oncogenes, and its knockdown suppresses tumor growth [26].	No
*MYC* (MYC proto-oncogene, bHLH transcription factor)	2.45	0.02	Nuclear phosphoprotein that plays a role in cell cycle progression, apoptosis, and cellular transformation. Its amplification is observed in several cancers [27].	No
*MAFF* (MAF bZIP transcription factor F)	3.79	0.02	Acts as a transcriptional activator or repressor, depending on its dimerization partner, and is associated with the cellular stress response [28].	No
*SOD3* (Superoxide dismutase 3)	2.47	0.02	Catalyzes the conversion of superoxide radicals into hydrogen peroxide and oxygen, thus protecting tissues from oxidative stress. Its role is context-dependent: in some cancers, downregulation is associated with progression, while in others, overexpression is linked to invasion [29].	No
*TNF* (Tumor necrosis factor)	0.16	0.02	Proinflammatory cytokine. Involved in proliferation, differentiation and apoptosis, among others [30].	Yes. Proposed role in VS hearing loss, proliferation, and contribution to tumor microenvironment.

Validation was divided into three phases: first phase consisted of studying the 10 samples which underwent transcriptomic analysis (recurrence = 4, non-recurrence = 6); second phase involved 11 additional cases selected to complete the previously established groups (recurrence = 2, non-recurrence = 9); third phase involved the study of a unique case with paired samples from both the initial surgery (VS60) and subsequent recurrence (VS90). 

### Statistical analysis

Statistical analyses were performed using GraphPad Prism 10.2.3 (Dotmatics). Expression data from RT-qPCR for each subgroup were log-transformed prior to analysis. Subgroup comparisons were conducted using t-test and one-way ANOVA. Survival analysis was performed using the Kaplan-Meier method and the Log-rank test; median follow-up time was calculated using the reverse Kaplan-Meier method. Either *p*-values or FDR-adjusted *p*-values < 0.05 were considered statistically significant.

## Results

### Patient cohort and clinical characteristics

A total of 22 patients and 23 tumor samples were included in the study: 7 from recurrent tumors and 16 from non-recurrent tumors (control group). Table 2 summarizes the patient demographic, genetic, and clinical characteristics. Mean age was 54 years (17–76), with a balanced sex distribution across groups. Most cases (*n* = 21) were sporadic VS, while 1 case (VS3) had NF2-SWN. This case was retained in the analysis, as recent studies suggest a similar immune and cellular gene expression profile between sporadic and NF2-SWN-associated tumors [31].

**Table 2:** Patient demographics, genetic profiles, and key clinical characteristics according to recurrence status and need for salvage surgery. Hearing classification follows the American Academy of Otolaryngology–Head and Neck Surgery (grades A–D) recommendation. NF2-SWN: *NF2*-related schwannomatosis, RT: radiotherapy, F: female, M: male, NA: not available

ID	Recurrence	Sex	Age	NF2- SWN	*NF2 *(blood)	*NF2 *(tissue)	Hearing	Previous RT
VS2	Yes	F	61	No	Negative	c.849delC	B	No
VS3	Yes	F	57	Yes	NA	NA	B	No
VS4	No	F	52	No	Negative	c.568_580delGCTTGGTACGC	C	No
VS20	Yes	M	45	No	Negative	c.658delA	A	No
VS21	Yes	F	56	No	NA	Negative	NA	No
VS22	No	F	43	No	Negative	Negative	B	No
VS24	No	M	52	No	NA	Negative	A	No
VS25	No	M	73	No	NA	Negative	D	No
VS27	No	M	35	No	NA	c.321delG; c.1002delG	C	No
VS30	Yes	M	69	No	Negative	c.1069_1070delGA	D	Yes
VS31	No	F	44	No	Negative	c.432 C > G	C	No
VS40	No	M	66	No	NA	NA	C	No
VS42	No	F	60	No	NA	NA	B	No
VS48	No	F	69	No	Negative	NA	C	No
VS52	Yes	M	17	No	Negative	c.459 C > G	D	Yes
VS55	No	F	53	No	Negative	NA	B	No
VS59	No	M	50	No	NA	NA	B	No
VS60	No	F	72	No	Negative	NA	D	No
VS61	No	F	76	No	Negative	c.555delA	D	No
VS62	No	M	48	No	Negative	NA	A	No
VS63	No	M	49	No	Negative	c.1613_1638del	B	No
VS71	No	M	59	No	Negative	NA	C	No
VS90	Yes	F	74	No	Negative	Negative	D	No

Among the 7 recurrent tumors, 10 prior surgeries had been performed: 3 GTR, 2 NTR, and 5 STR. Patients requiring three surgeries had previously undergone GTR (VS2) and STR (VS20, VS52). One patient underwent both primary (VS60) and subsequent salvage surgery (VS90) during the study period. 

### Recurrent tumors exhibit distinct transcriptomic profiles

**Fig. 1:**
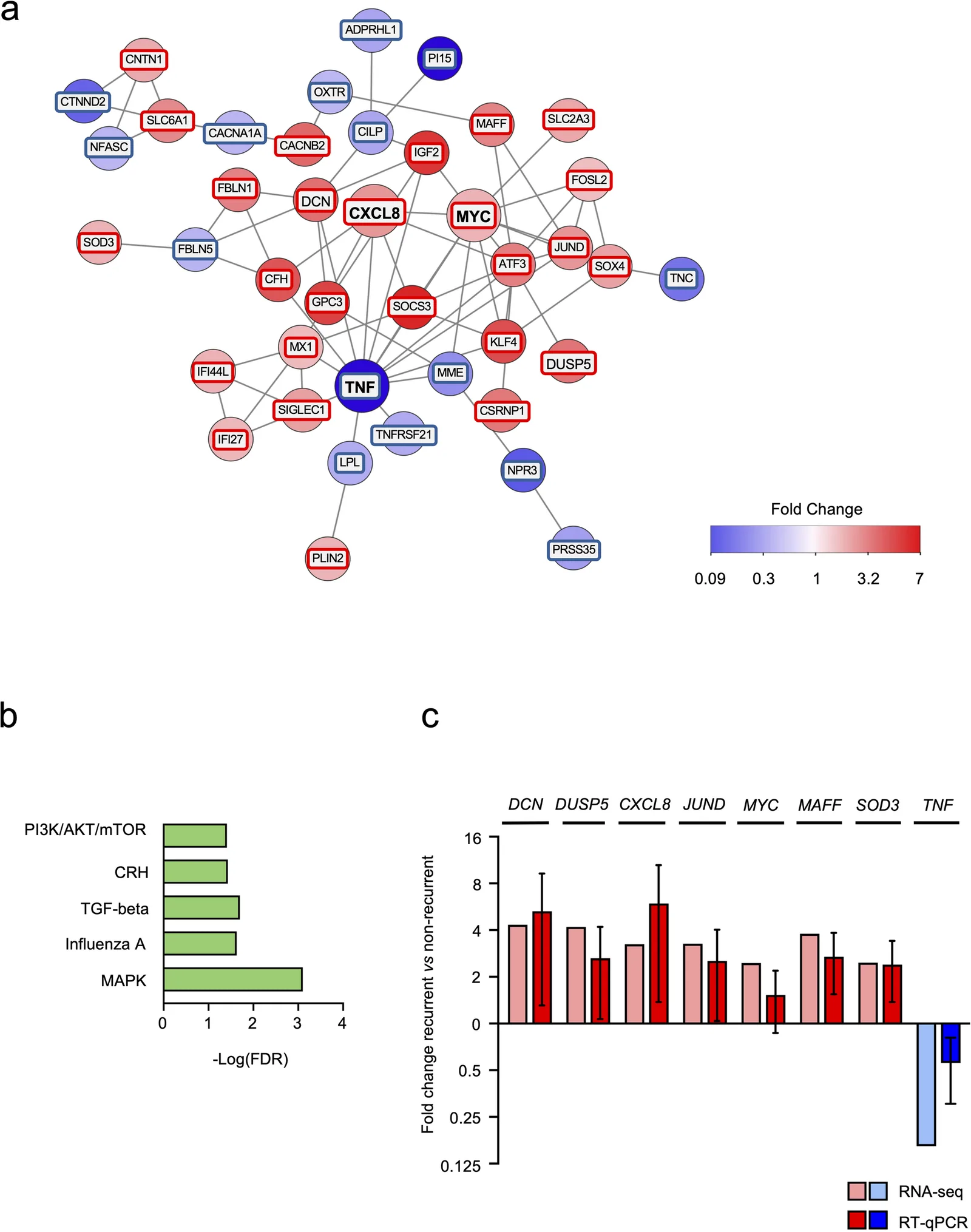
Differential gene expression in recurrent vestibular schwannomas based on RNA sequencing.** a** Protein–protein interaction network of differentially expressed genes from a sample set of *n* = 10, created with Cytoscape. Node color intensity indicates the magnitude of fold change in gene expression, as shown in the legend. **b** Enriched pathways identified by STRING functional enrichment analysis. Bars represent -Log of the false discovery rate (FDR). Pathways with FDR < 0.05 and strongest clinical relevance are shown. **c** Representation of RNA-Seq results (lighter bars) and RT-qPCR results (darker bars) of these *n* = 10 VS samples, as indicated in the Methods section. Individual bars represent the mean expression level of the indicated gene in the recurrence group relative to the non-recurrence group. For RT-qPCR, fold change was calculated using the 2^−ΔΔCt^ method and normalized to *HPRT1* expression. Error bars indicate the standard error of the mean of the fold change across individual tumor samples (each measured in technical triplicate)

RNA-Seq was performed on the initial 10 samples (4 recurrent, 6 non-recurrent). Comparison between recurrent and non-recurrent tumors identified 81 DEGs. Protein-protein interaction analysis revealed key molecular nodes, including *TNF*,* CXCL8*, and *MYC* **(**Fig. 1a; Table 1**).**

Pathway enrichment analysis highlighted significant involvement of the MAPK (FDR 7.9 E-4) and the PI3K/AKT/mTOR (FDR 0.038) pathways **(**Fig. 1b**)**. The MAPK pathway is known to contribute to the pathogenesis of peripheral nerve sheath tumors [32], and both the MAPK and the PI3K/AKT pathways are suppressed by merlin, the tumor suppressor protein commonly defective in VS [33]. 

### Identified candidate genes for recurrence were validated by RT-qPC

Based on transcriptomic findings, eight genes were selected for further validation through RT-qPCR: *DCN* (FC 4.34); *DUSP5* (FC 4.20); *CXCL8* (FC 3.25); *JUND* (FC 3.27); *MYC* (FC 2.45); *MAFF* (FC 3.79); *SOD3* (FC 2.47), and *TNF* (FC 0.16).

Validation was performed in three phases:

Phase 1) RT-qPCR on the original 10 RNA-Seq samples confirmed expression trends consistent with transcriptomic results (Fig. 1c**).**

**Fig. 2:**
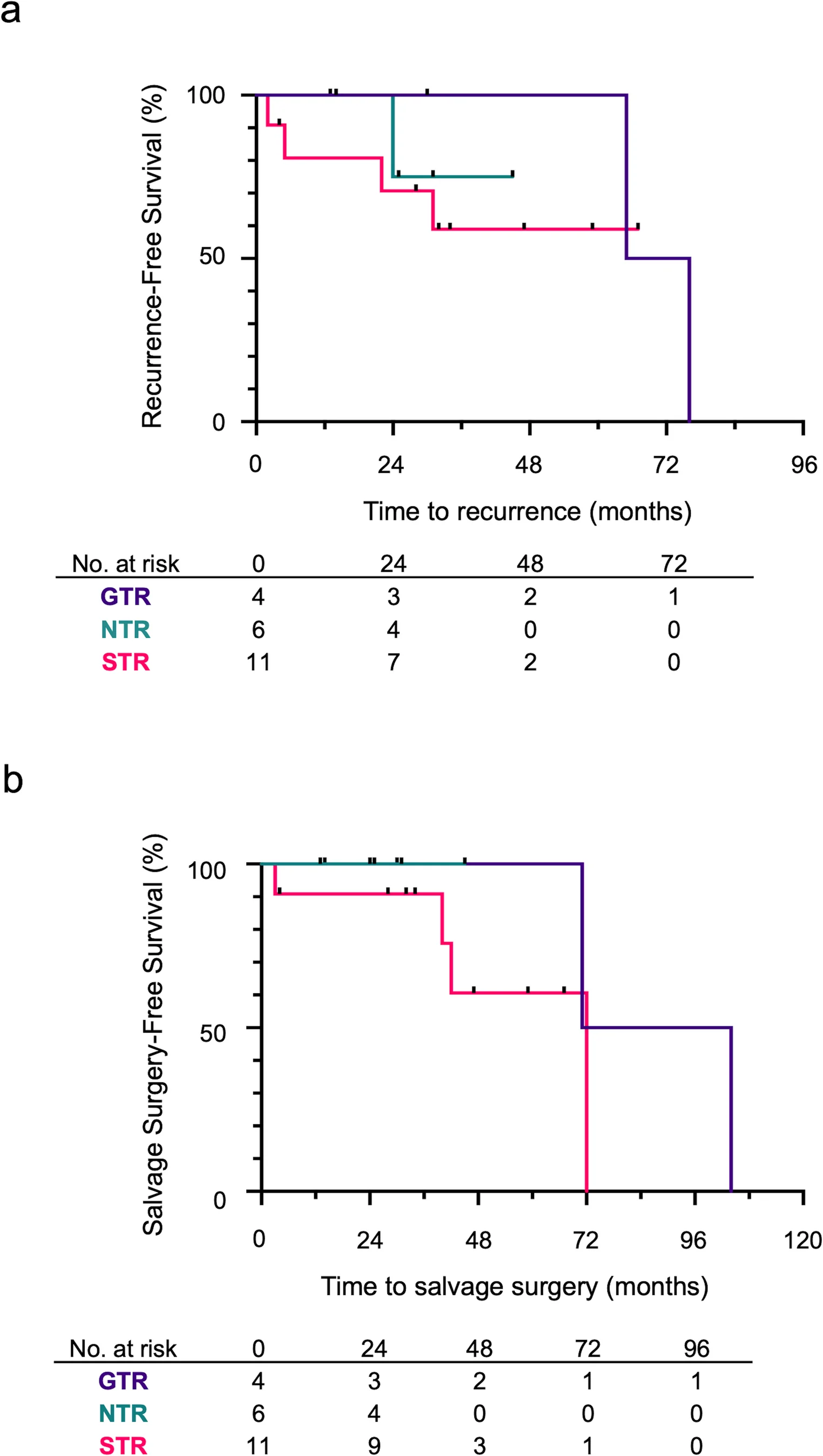
Kaplan–Meier survival analysis based on extent of resection.** a** Recurrence-free survival stratified by extent of resection. **b** Salvage surgery-free survival was stratified by the extent of resection. No statistically significant differences were observed between groups (log-rank test, *p* > 0.05). GTR: Gross Total Resection (purple lines); NTR: Near-Total Resection (green lines); STR: Subtotal Resection (pink lines)

Phase 2) 11 different samples (2 recurrent, 9 non-recurrent) were analyzed, bringing the total to 21 samples with 6 recurrent cases. We performed time-to-event analysis to study the influence of EOR in our cohort. The median total follow-up was 34 months (range, 4-328 months; 95% CI 25–67 months), with one patient having a very short follow-up due to medical complications after surgery. Patients with GTR had both longer RFS and SSFS, though differences were not statistically significant **(**Fig. 2**).**

#### Gene expression trends correlate with tumor aggressiveness

**Fig. 3:**
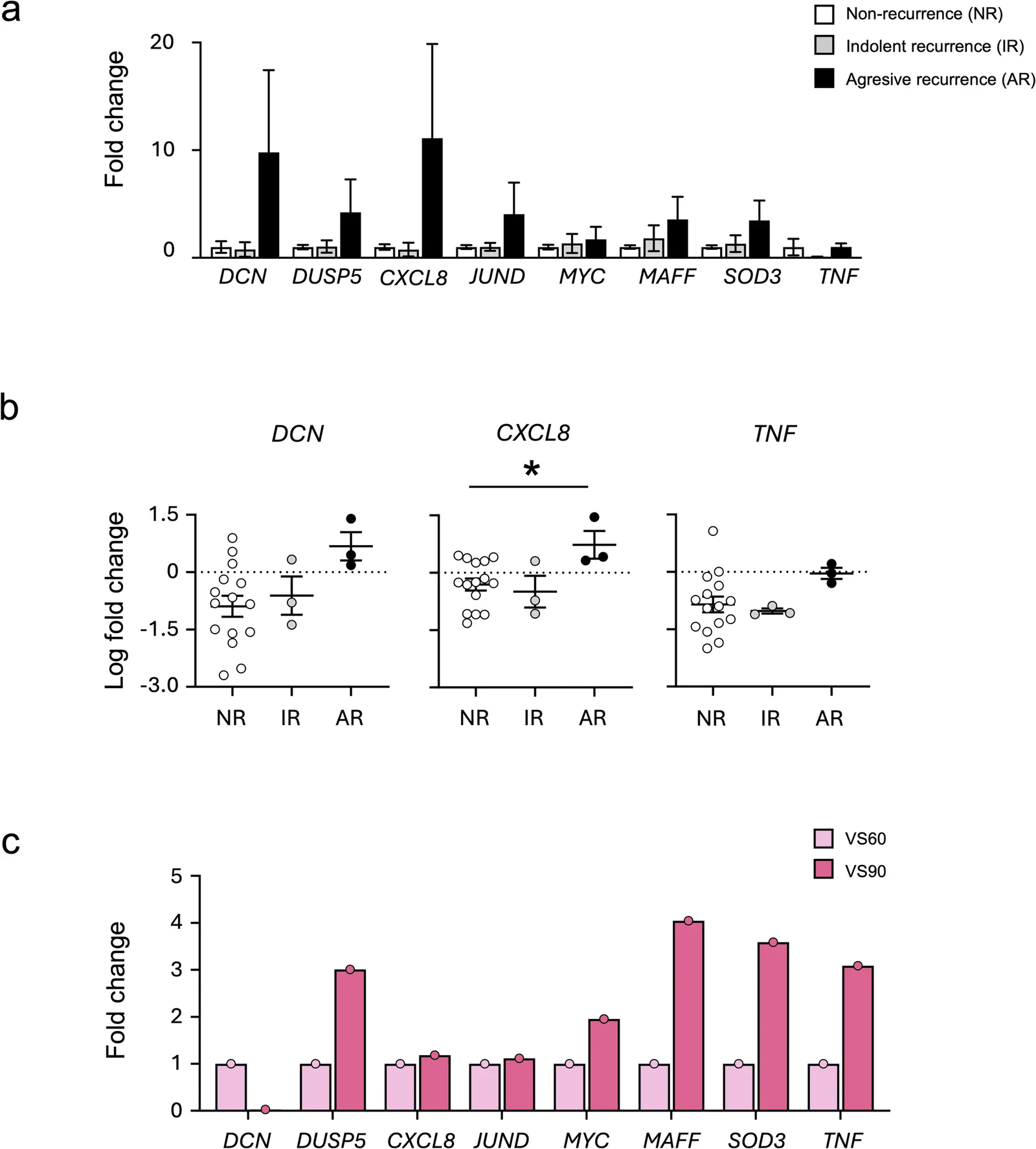
RT-qPCR validation of candidate genes.** a** Relative expression of the indicated genes in both recurrence subgroups. Each bar represents the mean gene expression level for each subgroup, compared to the non-recurrence subgroup. Relative expression was calculated using the 2^–ΔΔCT^ method, normalized to *HPRT1* as the reference gene. **b** Comparison of the expression levels of *DCN*, *CXCL8* and *TNF* in the three subgroups by using one-way ANOVA. Each data point represents the mean of three technical replicates per sample. Error bars indicate the mean ± standard error of the mean. *p ≤ 0.05; **p ≤ 0.01. NR: Non-Recurrence, IR: Indolent Recurrence, AR: Aggressive Recurrence. **c** *DCN*, *DUSP5*, *CXCL8*, *JUND*, *MYC*, *MAFF*, *SOD3* and *TNF* expression levels in two samples taken from a single patient whose tumor showed aggressive behavior after a subtotal resection. VS90 and VS60 codes correspond to recurrence and primary tumor samples, respectively. Bars represent mean expression levels in the recurrence sample relative to the primary sample. Relative expression was calculated using the 2^–ΔΔCT^ method with *HPRT1* as the reference gene

Across the aggressive subgroup, a trend toward higher expression levels was observed across all genes studied **(**Fig. 3a**).** Among the genes showing the most pronounced differences between subgroups, *DCN*, *CXCL8*, and *TNF* were notable. When comparing NR, IR, and AR subgroups, *CXCL8* expression was significantly elevated in the AR subgroup relative to NR. Comparing both subgroups individually, *TNF* reached statistical significance. Although overall *TNF* expression levels were low across all groups, AR showed higher *TNF* expression compared to IR.

Phase 3) Paired analysis of samples from a single patient (VS90 vs. VS60) revealed increased expression of *DUSP5*,* TNF*,* MAFF*, and *SOD3* in the recurrence sample, while *CXCL8*,* JUND*, and *MYC* remained stable, and decreased expression of *DCN*. 

#### Sensitivity analysis

We performed a sensitivity analysis excluding the NF2-SWN patient (VS3) from the cohort and re-evaluated the expression of *DCN*, *CXCL8* and *TNF* across the three groups. Since the exclusion of this patient reduced the AR subgroup to *n* = 2, non-parametric tests were applied (Mann-Whitney and Kruskal-Wallis tests). The direction of the effect was maintained for all three genes, which continued to show higher expression in the AR subgroup relative to NR and IR subgroups. However, statistical significance was not preserved for *CXCL8* when comparing NR, IR and AR subgroups (*p* = 0.11); nor for *TNF* when comparing NR to IR (*p* = 0.2). This finding is consistent with the markedly reduced statistical power resulting from the small size of the AR subgroup.

## Discussion

### Main findings

This study identifies a distinct molecular profile in patients with aggressive VS recurrence, a clinical phenotype that is not well characterized. Transcriptomic analysis revealed several DEGs in the recurrence group, including *DCN*, *DUSP5*, *CXCL8*, *JUND*, *MYC*, *MAFF*, *SOD3*, and *TNF*, with enrichment of the MAPK and the PI3K/AKT/mTOR pathways. Notably, *TNF* and *CXCL8* showed the highest significance in the aggressive subgroup, suggesting a potential role as biomarkers and therapeutic targets. 

### Extent of resection and recurrence

Most literature on VS recurrence consists of retrospective series emphasizing clinical variables, particularly EOR. STR is associated with the highest recurrence risk in some studies [9, 10]. However, EOR is influenced by different factors, including tumor vascularization and adherence to vital structures, which can vary considerably. In NF2-SWN, for instance, tumors tend to adhere more tightly to surrounding tissues than sporadic cases, increasing the risk of facial nerve injury and leading to a higher likelihood of partial resections [34].

Our survival analysis revealed no significant differences in RFS or SSFS between NTR and STR, contrasting with previous reports. Moreover, the NF2-SWN patient in our cohort underwent complete tumor removal and experienced recurrence, meaning that GTR can also be attempted in these cases, and that recurrence risk might be determined by factors other than EOR. 

### Genetic influence: *NF2* status

NF2-SWN is known to have a strong correlation between genotype and phenotype; in fact, a Genetic Severity Score has been proposed to stratify the clinical risk [35]. In contrast, in sporadic VS, this correlation is less clear. Previous studies suggest that copy-neutral loss of heterozygosity and mutations in other loci may correlate with aggressive behavior [36], while others have failed to establish a genotype-phenotype association [37].

*NF2* status was identified in over 60% of our samples **(**Table 2**).** Among recurrent cases, four harbored truncating deletions, while two were negative (one IR and the other, our single case-control patient). In the NR group, mutations ranged from early truncating to milder variants. Future assessments of our study group will clarify whether patients harboring a severe mutation exhibit a more aggressive disease evolution. 

### Inflammation and angiogenesis

Inflammation and angiogenesis are known to correlate with tumor growth [15]. In this study, *TNF* and *CXCL8* emerged as key differentially expressed genes in recurrent tumors. *TNF* expression was very low in the recurrence group, particularly in the IR subgroup, contrasting with higher expression in the AR subgroup. Furthermore, single-patient analysis revealed increased *TNF* expression in the recurrence sample compared to the primary tumor. TNFα has been extensively studied in VS, particularly in relation to hearing loss [30, 38] and tumor growth, through NF-κB signaling [30]. In fact, in vitro studies have shown that anti-inflammatory drugs can reduce VS cellular proliferation [39]. These findings support the role of inflammation in the development and aggressiveness of recurrence and suggest that dynamic changes in *TNF* expression could represent a potential prognostic marker, warranting validation in future studies with independent clinical cohorts and longitudinal follow-up. Therefore, monitoring serum TNFα levels during follow-up could aid in predicting tumor growth or recurrence, and further research should evaluate the effects of available targeted therapies, such as infliximab, etanercept, or adalimumab.

The vascular endothelial growth factor (VEGF) is the central mediator of angiogenesis. Bevacizumab, an anti-VEGF drug, has shown consistent effectiveness in terms of tumor shrinkage and symptom improvement in NF2-SWN patients [40, 41] and even in a sporadic VS patient [42]. It remains the only medical treatment endorsed by the Congress of Neurological Surgeons for NF2-SWN [43], though it is not FDA-approved for this indication.

The role of IL-8 in VS has not been previously characterized; however, its effects on the tumor microenvironment are well documented, including stimulation of VEGF production [24, 44]. Therefore, we hypothesize that AR might also respond to Bevacizumab. Additionally, investigational IL-8-targeting drugs currently in clinical development [44] may represent potential therapeutic options for VS cases exhibiting this molecular signature.

## Limitations

First, the EOR was assessed intraoperatively. Although this is the most used method in clinical practice, discrepancies between surgical and radiographic evaluations have been reported [9, 45].

Second, there was a methodological shift in genetic testing during the study period. Prior to 2022, a targeted panel was used; thereafter, Whole Exome Sequencing was adopted. As Whole Exome Sequencing enables broader detection of pathogenic variants, its absence in earlier cases may limit genotype-phenotype correlations.

Third, the sample size was relatively small, particularly in the recurrence subgroup. This limitation stems from the fact that VS is a rare disease, and recurrence is infrequent. Nevertheless, the small sample size restricts the statistical power of subgroup analyses and may affect the generalizability of our findings. The inclusion of an NF2-SWN case, although justified by emerging evidence of shared molecular features with sporadic VS, introduces additional heterogeneity into the cohort.

Finally, while our transcriptomic and RT-qPCR analyses revealed promising molecular candidates, functional validation of these genes and pathways was beyond the scope of this study. Further in vitro and in vivo studies are needed to confirm their roles in tumor progression and therapeutic response.

## Conclusions

VS exhibit a broad spectrum of clinical features, ranging from indolent lesions discovered incidentally to aggressive tumors that may put the life of the patient at risk. Despite recent advances, there are currently no reliable molecular predictors to guide therapeutic decision-making. Moreover, genetic alterations, such as *NF2* mutations, do not consistently correlate with clinical behavior, limiting their prognostic utility.

This study identified a distinct molecular signature associated with tumor recurrence following surgical intervention. Differentially expressed genes in recurrent tumors requiring salvage surgery included *DCN*, *DUSP5*, *CXCL8*, *JUND*, *MYC*, *MAFF*, *SOD3*, and *TNF*, with enrichment of the MAPK and the PI3K/AKT/mTOR pathways, known to play critical roles in malignant peripheral nerve sheath tumor biology and modulated by merlin, the tumor suppressor protein encoded by the *NF2* gene.

Validation through RT-qPCR confirmed significantly elevated expression of *CXCL8* and *TNF* in tumors exhibiting aggressive recurrence and requiring early salvage surgery. These findings suggest that TNF modulators and IL-8 inhibitors may represent promising therapeutic strategies for patients with this molecular profile.

Given the potential for recurrence and the morbidity associated with reintervention, a more precise molecular characterization of VS is of great clinical relevance. It could enable risk stratification and personalized treatment approaches and support closer surveillance in high-risk patients. Future studies should focus on expanding sample size, integrating longitudinal data, and exploring the functional roles of these molecular targets to translate these findings into clinical practice.

## Data Availability

RNA-seq data are available through the open-access institutional repository for Consejo Superior de Investigaciones Científicas (CSIC): https://doi.org/10.20350/DIGITALCSIC/17249. De-identified clinical data may be obtained upon reasonable request from the corresponding author.
